# Rock music improvisation shows increased activity in Broca’s area and its right hemisphere homologue related to spontaneous creativity

**DOI:** 10.1186/s13104-024-06727-6

**Published:** 2024-03-03

**Authors:** Atsumichi Tachibana, J. Adam Noah, Yumie Ono, Shun Irie, Muneto Tatsumoto, Daisuke Taguchi, Nobuko Tokuda, Shuichi Ueda

**Affiliations:** 1 Department of Anatomy, Dokkyo Medical University, Mibu, Tochigi Japan; 2 Department of Psychiatry, Yale School of Medicine, New Haven, CT USA; 3 Department of Electronics and Bioinformatics, Meiji University, Kawasaki, Kanagawa Japan; 4 Division for Smart Healthcare Research, Dokkyo Medical University, Mibu, Tochigi Japan; 5 Medical Safety Management Center, Dokkyo Medical University Hospital, Mibu, Tochigi Japan; 6 Department of Judo Therapy, Faculty of Medical Technology, Teikyo University, Utsunomiya, Tochigi Japan; 7 COSUMOPIA, Healthcare Facilities for the Elderly Requiring Long-Term Care, Mito, Ibaraki Japan

**Keywords:** Creativity, Broca’s area, Improvisation, Brodmann area 46, Functional near-infrared spectroscopy, Motor control, Rock music

## Abstract

**Objective:**

The neural correlates of creativity are not well understood. Using an improvised guitar task, we investigated the role of Broca's area during spontaneous creativity, regardless of individual skills, experience, or subjective feelings.

**Results:**

Twenty guitarists performed improvised and formulaic blues rock sequences while hemodynamic responses were recorded using functional near-infrared spectroscopy. We identified a new significant response in Broca’s area (Brodmann area [BA] 45L) and its right hemisphere homologue during improvised playing but not during formulaic playing. Our results indicate that bilateral BA45 activity is common during creative processes that involve improvisation across all participants, regardless of subjective feelings, skill, age, difficulty, history, or amount of practice. While our previous results demonstrated that the modulation of the neural network according to the subjectively experienced level of creativity relied on the degree of deactivation in BA46L, our current results independently show a common concurrent activity in BA45 in all participants. We suggest that this is related to the sustained execution of improvisation in “motor control,” analogous to motor planning in speech control.

**Supplementary Information:**

The online version contains supplementary material available at 10.1186/s13104-024-06727-6.

## Introduction

Creativity, widely used in daily life, has been suggested to be associated with higher-order brain function [1–4], but as one of the most complex human behaviors, the neural mechanisms associated with the expression of creativity remain unclear.

During musical improvisation as an expression of creativity, language-related cortical areas, such as Broca’s area and its right hemisphere homologue, are bilaterally activated [5]. The authors Donnay et al. concluded that such neural mechanisms are activated by interactive musical improvisation of expert jazz musicians and that musical discourse engages language-related brain areas specialized in syntax processing not contingent upon semantic processing. They suggest that neural regions for syntactic processing are not domain-specific for language and instead show domain-general activity correlated with multiple types of communication. As Donnay et al. studied only professional musicians, this activity in Broca’s area may reflect learned or trained behavior not present in beginners or amateurs [5].

In the present study, we aimed to determine whether Broca’s area shows increased activity during creative vs. formulaic rock guitar play across participants with widely varying skill sets, choosing guitarists with diverse musicianship, guitar play history, amount of daily practice, and age. We hypothesized that neural responses in the inferior frontal gyrus, including Broca’s area, show common increased and sustained activity during improvisation compared to formulaic scale play, independent of skill or background, as the common cortical regions are related to spontaneous creativity. It may be argued that activity differences between improvised vs. formulaic performance are similar to those between dynamic, dyadic conversations vs. overt monologues in which the speaker does not need to communicate with others [6]. As a clue to explore such differences, this research contributes to our understanding of how language production centers contribute to improvisation and creativity in a more general sense.

A previous study has linked specific patterns of neural activity in the prefrontal cortex (PFC) to creativity during improvised jazz piano performances using functional magnetic resonance imaging but enrolled only professional and semi-professional musicians [7]. While these studies contributed to a fundamental theory regarding the relationship between the medial frontal lobe (MFL) and dorsolateral prefrontal cortex (DLPFC) during improvisation, they did not show that these neural activities are generalizable to a wider population. The results of our previous report [8] imply that when studying creativity, it is important to not only investigate professional and trained populations with years of expertise but also to explore these same circuits in non-experts using tasks constructed by simple popular musical theory, such as rock music, that can be performed by a wider audience. Using a more diverse sample can not only increase sample size and power but allow the investigation of aspects of learning related to creativity that may no longer be present in musicians who have played professionally for most of their lives. By studying beginners and amateurs as well as professionals, it may be possible to further understand a “generalizable process of creativity” and the neural circuits that underlie this process.

Using functional near-infrared spectroscopy (fNIRS), we assessed in this previous study the hemodynamic responses in the PFC during improvised and formulaic play on an electric guitar with a background music (BGM) track [8]. The results comparing formulaic with improvised sequences support a framework previously proposed by other researchers [7], documenting increased activity in the MFL with decreased activity in the DLPFC. In addition to these specific neural responses, we found a significant negative correlation between subjective feelings of improvisational performance and modulation in Brodmann area (BA) 46 of the left DLPFC. In other words, participants who felt more subjective creativity while improvising tended to suppress BA46L activation. Importantly, this finding was present during improvisation by musicians across different skill sets. On the other hand, since all participants played the improvised performance task differently, they all might use a common neural strategy that does not include BA46L. Of course, this strategy should differ from that used during formulaic performance.

Given our hypothesis, we explored these additional neural mechanisms using the methods of our previous report [8].

## Methods

The study was conducted in accordance with the principles of the Declaration of Helsinki. The Dokkyo Medical University Institutional Review Board examined and authorized the research involving humans (approval number: 26002). All participants (20 men including three professional musicians, age 19–63 years) provided written informed consent prior to enrollment.

Data from brain regions that had not been previously discussed [8] were analyzed. The fNIRS data were obtained from the same 20 participants using the methods of the previous report, and data analysis was performed under the same task conditions. The participants were required to perform the tasks without making facial expressions, including mouth movements. As reported, the paired *t*-test revealed no significant behavioral differences in the total number of strings picked during improvised play (Improv) and playing predetermined formulaic music scales (Formulaic) conditions [8]. To explore correlational relationships of Broca’s area and its right hemisphere homologue with improvised or formulaic guitar play behavior, each participant was asked to answer immediately after the fNIRS scan a questionnaire comprising two visual analogue scales and three questions regarding experience/historical information, as previously described [8]. The questionnaire included the following five items: Q1, F-I value (subjective degree of formulaic-improvised feeling during the Improv task); Q2, difficulty (subjective feeling of task difficulty); Q3, age of the participant; Q4, history (number of years the participant had been playing guitar); and Q5, practice (number of hours of daily practice). This methodology and the behavioral data are detailed elsewhere [8].

## Results

### Event-triggered averages of hemodynamic signals for tasks vs. baseline

**Fig. 1:**
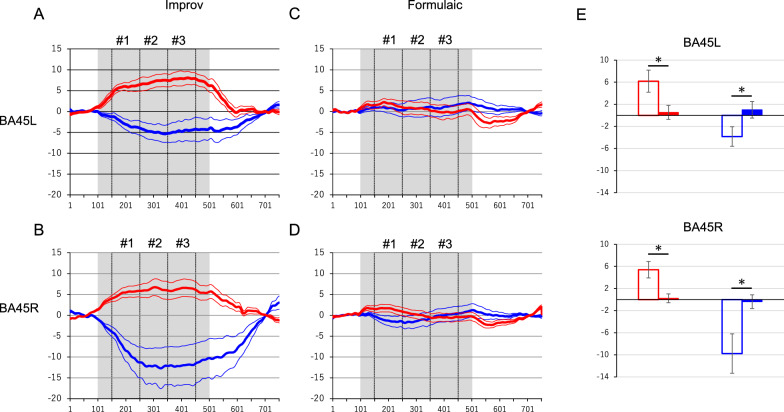
**A**–**D** Plots of the averaged activation patterns (± SEM) for oxyHb (red) and deoxyHb (blue) in BA45L and BA45R during the Improv and Formulaic tasks. The gray areas and three vertical dashed lines indicate the task duration and each bin (#1–3) divided by 10 s in the respective graphs. **E** Result of the comparisons between Improv (open bar) and Formulaic (closed bar) tasks. *p < 0.05

Within Broca’s area (BA45L) and its right hemisphere homologue (BA45R), we compared oxyhemoglobin (oxyHb) and deoxyhemoglobin (deoxyHb) responses between Improv and Formulaic conditions (Fig. 1A–D). Interestingly, the activities during Improv showed hemodynamic responses including significantly increased oxyHb and decreased deoxyHb values (p < 0.05) compared to those during Formulaic (Fig. 1E).

**Fig. 2:**
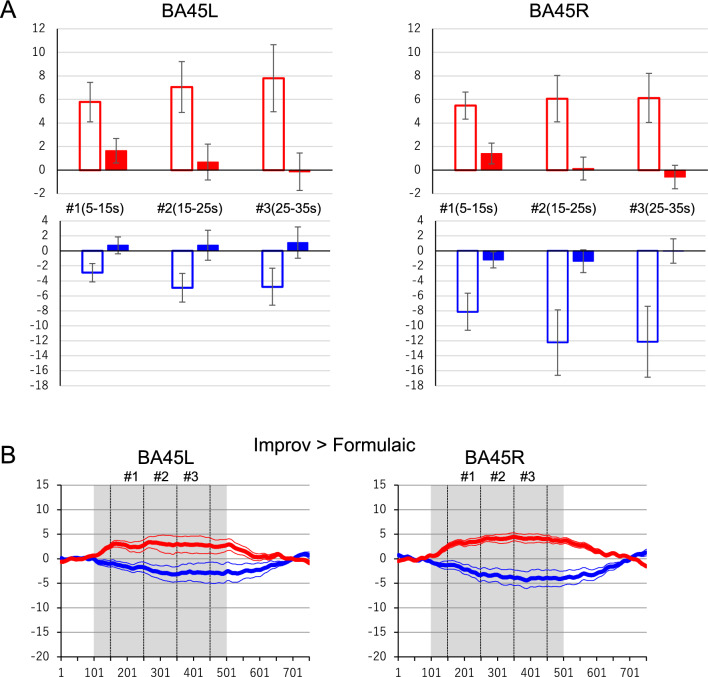
**A** Averaged hemodynamic responses in BA45 in each 10-s bin during Improv and Formulaic task conditions. The activation magnitude in the analysis windows of each 10-s bin (Fig. 1A–D, #1–3) and their 1-SEM range are shown during Improv (open bar) and Formulaic (closed bar) tasks in BA45R and BA45L (red for oxyHb and blue for deoxyHb). (**B**) Averaged time-courses of hemodynamic responses by subtraction analysis from Improv to Formulaic of BA45 in the left and right hemispheres. Red and blue lines show oxyHb and deoxyHb responses, respectively. Thick and thin traces represent the mean response amplitude and its 1-SEM range, respectively. The gray areas and three vertical dashed lines indicate the task duration and each bin (#1–3) divided by 10 s, respectively

Figure 2A demonstrates that, apart from oxyHb in BA45L, hemodynamic responses during Improv (open bars) were significantly larger than those during Formulaic (closed bars; p < 0.05, Holm’s test) conditions in the comparison of these two tasks. Two-way repeated-measure ANOVAs indicated a significant interaction in all brain regions for both oxyHb and deoxyHb (p < 0.05, see Additional file 1). Moreover, significant activity compared to baseline was observed for both oxyHb and deoxyHb responses in the Improv, but not Formulaic, task (p < 0.05, Dunnett’s test).

### Event-triggered average contrast of Improv > Formulaic tasks

The traces in Fig. 2B indicate the event-triggered average plot (± SEM) of the results of the contrast Improv > Formulaic from BA45L/R. All averaged contrasted responses demonstrated that the task-related oxyHb and deoxyHb responses were significantly increased and decreased, respectively, compared to baseline (p < 0.05).

### Correlation between questionnaire responses and hemodynamic signals

Variables of skill, history, age, and subjective feelings were also investigated regarding their influence on guitar tasks and neural activity. Correlations between the subjective creative index (F-I value), difficulty, history, age, and practice in addition to hemodynamic contrasts were calculated (Figs. 1A–D and 2B). No correlations were found between oxyHb and deoxyHb signals in BA45L/R with respect to F-I value, difficulty, history, age, or practice (p < 0.05; see Additional file 2).

## Discussion

This is only the second study to explore correlations between brain dynamics and creativity in the production of rock music played on a guitar. Here, we hypothesized that regions in the cortex may display larger responses during “improvised execution” compared to “formulaic execution” regardless of individual variability in skill or behavior due to the increased spontaneity in the improvised compared to the formulaic task. The results indicated common bilateral activation of oxyHb and deactivation of deoxyHb signals in language areas, including Broca’s area (BA45L) and its right hemisphere homologue (BA45R) in all participants, in the improvised task (Fig. 1). The improvised performance elicited a significant change in hemodynamic activity in BA45L/R compared to the formulaic performance. The magnitudes of activation in the analysis windows of each 10-s bin (#1–3) during Improv (open bar) and Formulaic (closed bar) tasks in BA45R and BA45L indicated a significant interaction in all brain regions and both oxyHb and deoxyHb signals (Fig. 2). The hemodynamic responses during the Improv task were significantly larger than those during the Formulaic task, except for oxyHb in BA45L. Furthermore, these hemodynamic responses showed no correlation with subjective feelings, difficulty, history, age, or amount of practice.

In our previous study, we found decreased left DLPFC (BA46L) activity in response to improvised guitar play representing a creative task, which was significantly correlated with subjective feelings of improvisational guitar performance as reported by the musicians [8]. These results suggest that subjective feelings of improvisational performance and decreased deoxyHb signals in BA46L are highly correlated with evoked creativity regardless of skill level.

Recently, Donnay et al. reported that musical discourse engages syntax-processing areas of the brain, including Broca’s area and its right hemisphere homologue, in a manner that is not contingent upon semantic processing [5]. They concluded that the neural regions for syntactic processing were not domain-specific for spoken language, but instead were domain-general across multiple forms of communication. Despite the strong conclusions, some limitations in their study must be addressed: all participants were highly proficient jazz pianists who possessed expert communication skills with other players in jam sessions, and the domain-general hypothesis was supported by an improvisation paradigm that featured only one session style (i.e., between two players with a background track). This style is different from solo improvised performances, where communication between individuals and the production of music are ongoing in real time. With multiple improvisational tasks complementing each other simultaneously, these processes may produce confounding effects in the communication centers of the brain.

The results of this study provide additional support for the domain-general hypothesis regarding activity in Broca’s area and its right hemisphere homologue. While activity in these areas is present in both improvised and formulaic tasks, we have shown that hemodynamic responses in BA45L/R in the improvised task are greater than those in the formulaic task during solo performances with BGM regardless of musical skill. We previously reported that deoxyHb in BA46L in only the last 10-s bin (#3) indicated a high correlation with the F-I value [8]. In contrast, we demonstrated here that the hemodynamic responses in BA45L/R in the improvised task were consistently greater than those in the formulaic task in each 10-s bin. In addition, hemodynamic responses were not correlated with F-I value, difficulty, history, practice, or age in these participants. This suggests that BA45L/R are common cortical regions related to spontaneous improvised execution across all players, regardless of subjective feelings of creativity, and are not confounded with domain-general communication between performers. Although Dhakal et al. previously reported the mechanisms of spontaneous creativity by musical improvisation—one of which related to Broca’s area involvement in evaluation processes and another one related to the translation of abstract information to motor commands—we additionally suggest that continuous hemodynamic activity in Broca’s area and its right hemisphere homologue must be needed for spontaneous, improvised execution in any player throughout the entire task as a common mechanisms [9].

**Fig. 3:**
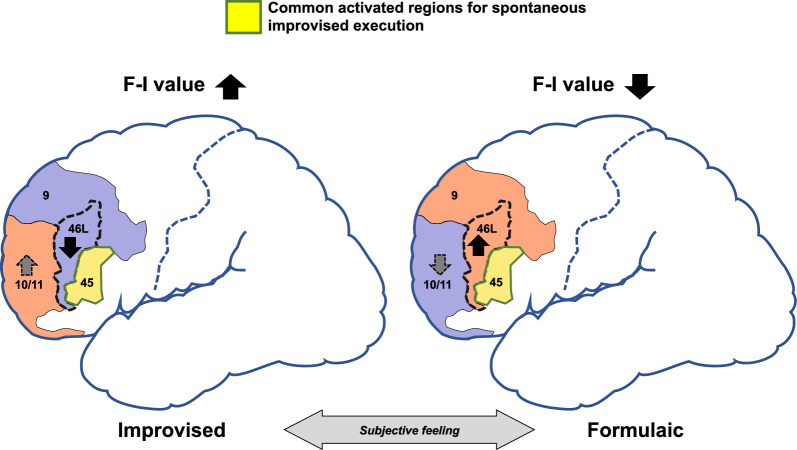
Schema of prefrontal relationships in relation to spontaneous creativity through musical improvisation. Prefrontal activation patterns are described as a function of subjective feeling. BA46L shows activation and deactivation, respectively, in conjunction with subjective feelings of improvisation vs. formulaic guitar play. BA10/11 is causally implicated in activation and deactivation, as shown by dashed arrows. BA45L/R represents commonly activated regions across participants regardless of skill, history, amount of daily practice, or age

The present study demonstrates that activity in BA45L/R is related to the neural circuitry of improvised content and the expression of creativity, rather than the results of Donnay et al. showing connectivity between the left and right hemispheres for domain-general communication with other performers [5]. This finding and those of our previous study suggest a neural model of spontaneous creativity [8]. The increased activity in the MFL and decreased activity in the DLPFC are related to the subjective feeling of improvised creativity. In addition, concurrent activities in Broca’s area and its right hemisphere homologue are related to and necessary for the sustained execution of improvised creative performance in motor control analogous to motor planning and control for speech (Fig. 3).

## Limitations

This study has some limitations regarding sample size and participant gender bias, the questionnaire categories for a superior correlation factor for spontaneous creativity, and the systemic artifact removal for the Hitachi 4000 fNIRS topography system. Details of these limitations were previously described [8].

### Supplementary Information

**Additional file 1.** Repeated measures ANOVAs of oxy- and deoxyHb in all brain regions.

**Additional file 2.** Correlations between BA45 and scores from a post-experimental questionnaire.

## Data Availability

The datasets generated and analyzed during the current study are available from the corresponding author upon reasonable request.

## Abbreviations

BA: Brodmann area
BGM: Background music
deoxyHb: Deoxyhemoglobin
DLPFC: Dorsolateral prefrontal cortex
fNIRS: Functional near-infrared spectroscopy
Formulaic: Playing predetermined music scales
Improv: Improvised play
MFL: Medial frontal lobe
oxyHb: Oxyhemoglobin
PFC: Prefrontal cortex
