# Supplementary material for: Rock music improvisation shows increased activity in Broca’s area and its right hemisphere homologue related to spontaneous creativity
Source: BMC Res Notes. 2024 Mar 3;17:61. doi: 10.1186/s13104-024-06727-6 (PMC10909250; doi:10.1186/s13104-024-06727-6)
Supplement: Supplementary file 1 — Additional file 1. Repeated measures ANOVAs of oxy- and deoxyHb in all brain regions. [file 13104_2024_6727_MOESM1_ESM.pdf]

Additional file 1 Repeated measures ANOVAs of oxy- and deoxyHb in all brain regions.

Factor	*df*	*F*	*p*	*Factor*	*df*	*F*	*p*
OxyHb in BA45L				OxyHb in BA45R			
Time	3	3.662	0.018	Time	3	5.987	0.001
Task	1	3.595	0.073	Task	1	10.89	0.004
Time × Task	3	3.776	0.015	Time × Task	3	9.271	<0.001
DeoxyHb in BA45L				DeoxyHb in BA45R			
Time	3	1.065	0.371	Time	3	6.007	0.001
Task	1	7.276	0.014	Task	1	6.792	0.017
Time × Task	3	5.274	0.003	Time × Task	3	6.160	0.001

Note: df: degrees of freedom, F: F-value, Hb: hemoglobin, p: p-value

Methods of statistical analysis in the effects of tasks and time scales on the hemodynamic response

To assess the effects of the two tasks and time scales on hemodynamic responses, we performed a repeated-measures ANOVA between the factors and the interaction. The dependent variables were hemodynamic responses (oxyHb or deoxyHb). The independent variables were set to the time scale (baseline and #1-3 analysis windows) and task (Improvise vs. Formulaic). When the interactions reached a level of significance ($\alpha = 0.05$), post hoc comparisons were performed for

each factor. For comparison across time scales, we used the Dunnett's test to assess the differences in the mean responses in the four analysis windows compared to baseline. To compare the tasks, we performed paired *t*-tests with Holm's correction. Group data are expressed as mean ± SE unless otherwise noted. All statistical analyses were performed using R version 4.1.0 (R Foundation for Statistical Computing, Vienna, Austria).
