# Supplementary material for: Rock music improvisation shows increased activity in Broca’s area and its right hemisphere homologue related to spontaneous creativity
Source: BMC Res Notes. 2024 Mar 3;17:61. doi: 10.1186/s13104-024-06727-6 (PMC10909250; doi:10.1186/s13104-024-06727-6)
Supplement: Supplementary file 2 — Additional file 2. Correlations between BA45 and scores from a post-experimental questionnaire. [file 13104_2024_6727_MOESM2_ESM.pdf]

Additional file 2 Correlations between BA45 and scores from a post-experimental questionnaire

BA45L	R value														
Signal	F-I value			Difficulty			History			Practice			Age		
	Improv	Form	Subtract	Improv	Form	Subtract	Improv	Form	Subtract	Improv	Form	Subtract	Imp	Form	Subtract
oxy	0.102	0.256	0.007	0.031	0.040	0.050	0.005	0.025	0.004	0.008	0.010	0.000	0.000	0.032	0.007
deoxy	0.000	0.057	0.002	0.021	0.001	0.009	0.152	0.001	0.109	0.018	0.018	0.052	0.150	0.000	0.138

BA45R	R value														
Signal	F-I value			Difficulty			History			Practice			Age		
	Improv	Form	Subtract	Improv	Form	Subtract	Improv	Form	Subtract	Improv	Form	Subtract	Imp	Form	Subtract
oxy	0.002	0.256	0.011	0.005	0.000	0.035	0.043	0.026	0.034	0.024	0.095	0.001	0.041	0.017	0.045
deoxy	0.033	0.052	0.0542	0.027	0.003	0.030	0.007	0.005	0.002	0.033	0.006	0.001	0.016	0.048	0.000

Note: No high correlation was observed ($r > 0.5$) in any condition, that is, Improv > Formulaic (Subtract), Improv > Baseline (Imp), and Formulaic > Baseline (Form) tasks.
